# Myopericytoma mimicking subcutaneous melanoma metastasis

**DOI:** 10.1007/s40477-024-00884-x

**Published:** 2024-04-18

**Authors:** Inés Oteiza-Rius, Ana Morelló-Vicente, Elisa María Gómez-González, Ane Carrera-Gabilondo, Francisco Javier García-Martínez

**Affiliations:** 1 Department of Dermatology, Clínica Universidad de Navarra, Pio XII 36, 31008 Pamplona, Spain; 2 Department of Dermatology, Clínica Universidad de Navarra, Madrid, Spain

**Keywords:** Sonography, Myoperycitoma, Melanoma

## Introduction

Myopericytomas (MPs) are uncommon benign tumors typically found in young women [1]. Till date, fewer than 200 cases have been reported in literature, reason why these tumors are easily misdiagnosed [2]. While histological examination is required for diagnostic confirmation, ultrasound is a highly useful imaging tool for diagnostic guidance and surgical management.

## Clinical case

**Fig. 1:**
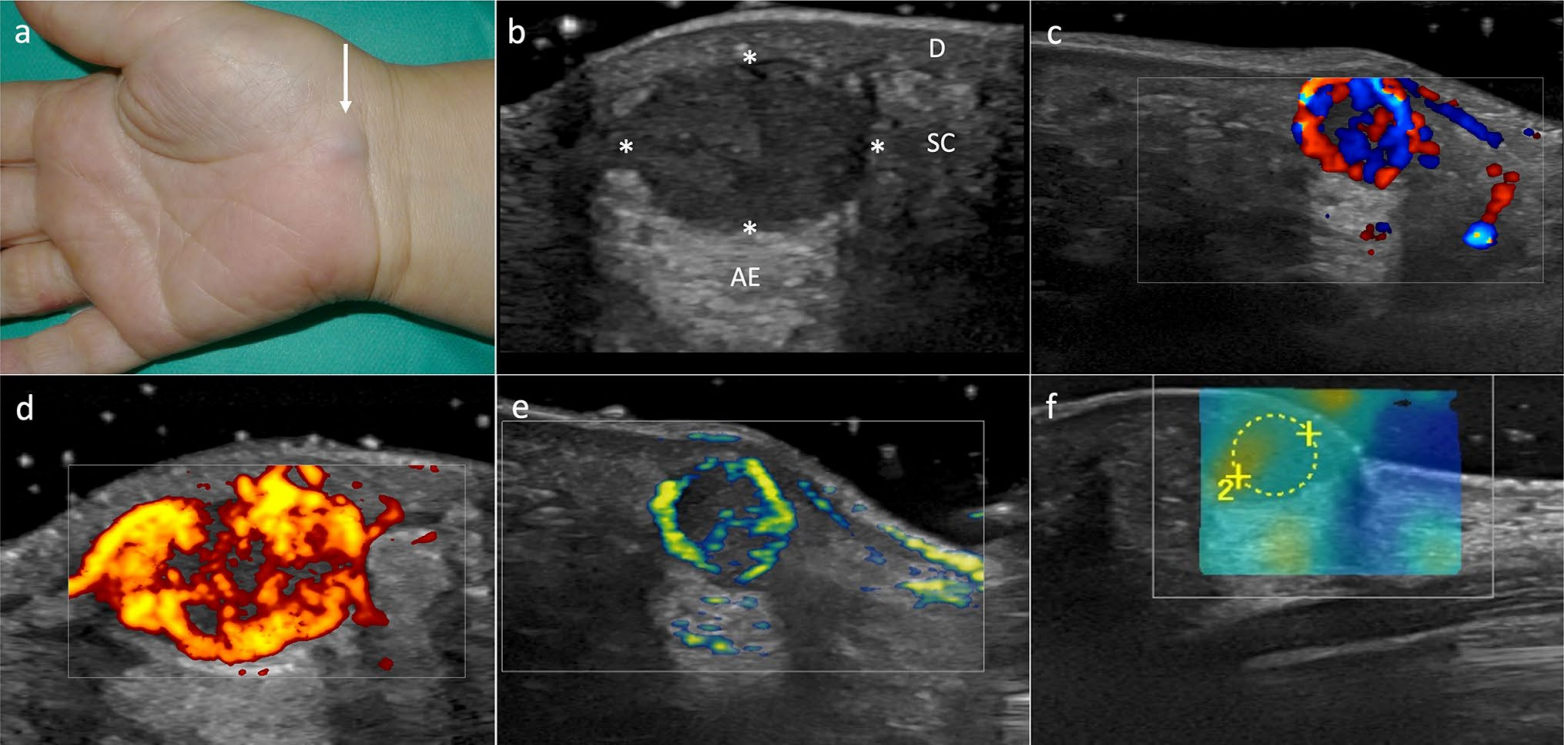
**a** Subcutaneous nodular lesion with a bluish discoloration located on the anterior region of the right wrist (arrow). **b**–**d** Sonographic study of the lesion. **b** A well-defined, non-encapsulated, hypoechoic, round dermal (D)-subcutaneous (SC) lesion (*) with posterior acoustic enhancement (AE). **c** Color Doppler, **d** power Doppler and **e** elastography studies

**Fig. 2:**
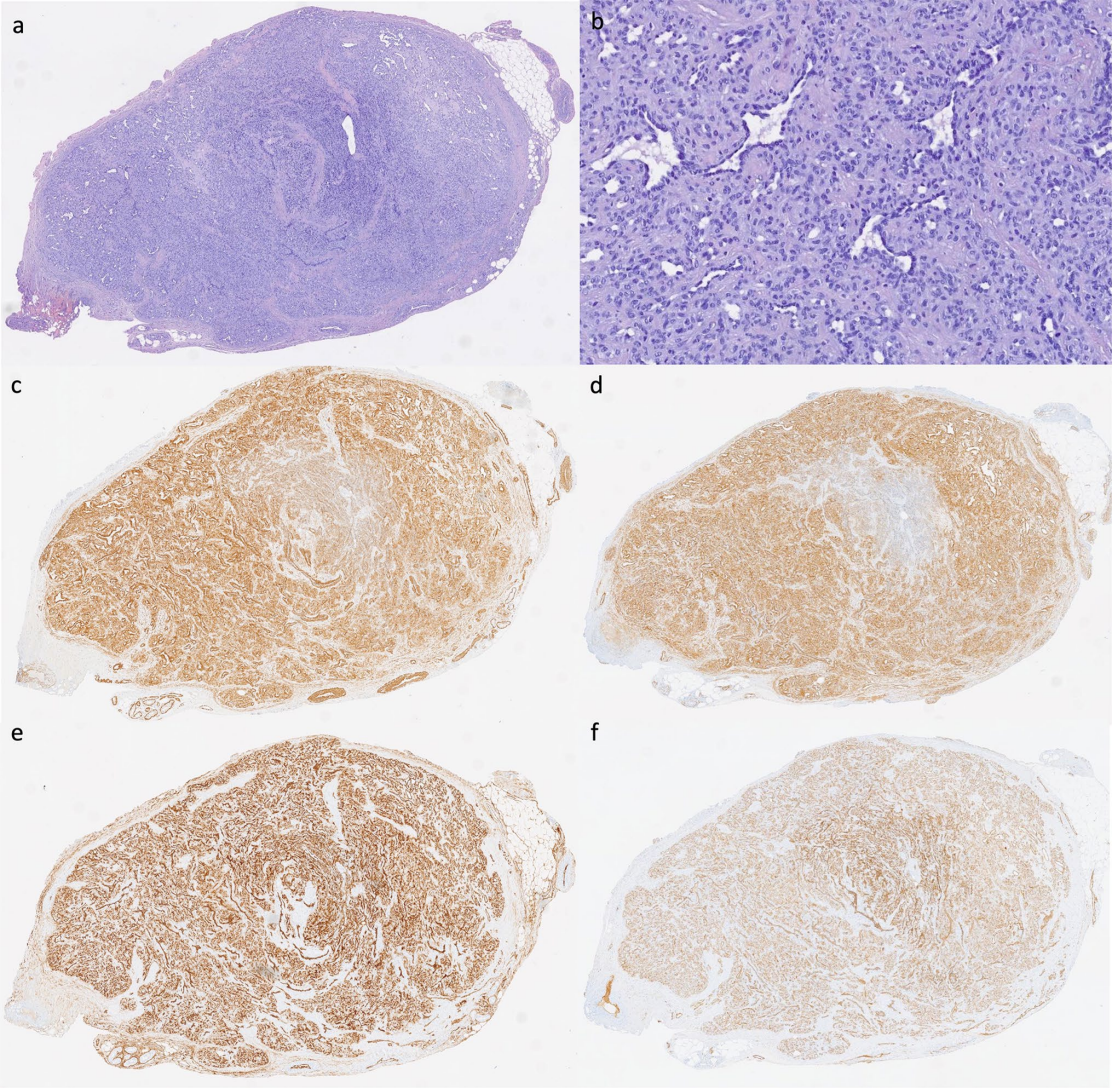
Dermatopathological study. **a** At lower power, histopathological images of the cutaneous lesion show a non-encapsulated nodular lesion with peripheral fibrosis (H-E, × 40). **b** High power image showing the presence of oval-shaped cells with eosinophilic cytoplasm, with a concentric vascular growth (HE, × 100). Immunohistochemical study showing positive results for smooth **c** muscle actin, caldesmon (**d**), CD34 (**e**), and CD31 (**f**), all consistent with the diagnosis of MP

We report the case of a 58-year-old woman with a history of vulvar melanoma with a Breslow’s thickness of 13 mm and involvement of left inguinal lymph nodes (T4BN2M0). At that moment, she was undergoing proton therapy targeting the regional pelvic lymph nodes and was under clinical and ultrasound follow up. The patient was referred to our Dermatology Department to rule out metastasis to due to a subcutaneous nodular lesion located on the anterior region of the right wrist, with a bluish discoloration that had initiated 6 weeks ago (Fig. 1a). Longitudinal and cross-sectional sonography scans with L10-22 MHz, L8-18i MHz and ML6-15 MHz linear probes showed in B-mode a well-defined, non-encapsulated, hypoechoic, round dermal-subcutaneous lesion with posterior acoustic enhancement (Fig. 1b). Color Doppler (Frequency 10 MHz, PRF: 1.9) (Fig. 1c), Power Doppler (Frequency 14.3 MHz, PRF 0.7) (Fig. 1d) and B-Flow mode (Frequency 8 MHz, PRF 0.6) (Fig. 1e) exhibited an intense peripheral ring-like vascularity suggestive of a vascular-origin tumor. Spectral Doppler (Frecuency 12.5 MHz, PRF 4.1) displayed a biphasic waveform with a peak systolic velocity of 7 cm/s and a resistive index of 0.68. In addition, shear wave elastography using the 6–15 MHz probe revealed a median value of 3.5 m/s and 31.71 kPa (Fig. 1f). Given the clinical context, it was decided to proceed with excision. Anatomopathological examination revealed a non-encapsulated nodular lesion with peripheral fibrosis and presence of oval-shaped cells with eosinophilic cytoplasm, demonstrating concentric vascular growth. Immunohistochemical staining was positive for smooth muscle actin, caldesmon, and CD34, all consistent with the diagnosis of MP (Fig. 2).

## Discussion

MPs are nodular lesions of slow growth, typically located in subcutaneous tissue of distal extremities [3]. These lesions are usually painless and well circumscribed. Anatomopathological examination can sometimes allow to differentiate them from clinically similar tumors such as myofibromas, glomus tumors, or angioleiomyomas [4]. MPs consist on a combination of solid cellular regions comprised of oval and spindle-shaped cells and vascular channels with prominent branches [5]. On contrary, myofibromas show a biphasic growth pattern and a myxoid stroma [6]. In the case of glomus tumors, they also present a perivascular arrangement of cells but are not concentrically arranged and they present a more centrally distributed vascularization [7]. Angioleiomyomas feature abundant vascular channels but can be differentiated from MPs as they usually present smooth-muscle fascicles, which stain positive for desmin. It has been suggested that the malignant potential of MPs is strongly associated with the depth of the tumor; however, only a limited number of cases have been reported [1]. Even though there have been reports on clinical and pathological characteristics of several MP cases, these tumors are still often misdiagnosed with metastasis or other malignant tumors [1]. Ultrasound has been proven to be a useful and precise tool for describing, locating, and treating several diseases in numerous studies [8, 9]. Moreover, taking into account that ultrasound scans should be considered a first-choice screening tool for a palpable mass, we believe that sonography studies could be determinant in the management of these lesions, to avoid unnecessary biopsies or tumor excisions. As it is seen in histopathological studies, ultrasonography shows well-defined, non-encapsulated, hypoechoic, round dermal-subcutaneous lesions with posterior acoustic enhancement. Color and Power Doppler help to determine the vascular pattern and differentiate it from other vascular-origin tumors. On the other hand, pulse wave Doppler usually exhibits the presence of low blood flow in intraparenchymal vessels with a moderate resistance. In addition, elastography can reveal the soft consistency of myopericytomas, which enables to discern them from other malignant tumors, such as metastasis.

## Conclusion

Cutaneous ultrasound in this patient with a history of metastatic melanoma enabled us to suggest alternative diagnoses and avoid assuming tumor progression, thereby improving management and surgical precision. While cutaneous ultrasound has proven to be highly relevant for the proper management of this patient; to date, the ultrasound characteristics of cutaneous MPs have not been described.

## Data Availability

Not applicable.

## References

[1] Dray MS, McCarthy SW, Palmer AA, Bonar SF (2006) PD Stalley, et al Myopericytoma: a unifying term for a spectrum of tumours that show overlapping features with myofibroma. A review of 14 cases. J Clin Pathol 59:67–73. 10.1136/jcp.2005.028704 16394283 10.1136/jcp.2005.028704 PMC1860256

[2] Boix-Vilanova J, Del Pozo Hernando LJ, Rodrigo Lara H, Corral- MO (2020) Distal digital myopericytoma: a dermoscopic case study. Actas Dermosifiliogr 111:338–341. 10.1016/j.adengl.2020.04.001 31627853 10.1016/j.ad.2018.09.015

[3] Wei PTM, You WTA, Yin TP et al (2022) Myopericytoma: a review of twenty-three cases over twelve years and a case report of a rare neoplasm. A J Dermatopathol 44(9):623–631. 10.1097/DAD.0000000000002130 10.1097/DAD.0000000000002130 35980090

[4] Cockburn CJK, Crene EJD, Cockburn WJ (2022) Pre-tibial myopericytoma: a case report. J Surg Case Rep 2(2):rjac21. 10.1093/jscr/rjac021 10.1093/jscr/rjac021 PMC8824466 35145630

[5] Phyu PA, Lynne JG, Meera M, Jag B (2015) Cutaneous myopericytoma: a report of 3 cases and review of the literature. Dermatopathology 2(1):9–14. 10.1159/000371875 27047931 10.1159/000371875 PMC4816429

[6] Choi JH, Ro JY (2018) Cutaneous spindle cell neoplasms: pattern-based diagnostic approach. Arch Pathol Lab Med 142(8):958–972. 10.5858/arpa.2018-0112-RA 30040457 10.5858/arpa.2018-0112-RA

[7] Mravic M, LaChaud G, Nguyen A, Scott MA, Dry SM, James AW (2015) Clinical and histopathological diagnosis of glomus tumor: an institutional experience of 138 cases. Int Surg Pathol 23(3):181–188. 10.1177/1066896914567330 10.1177/1066896914567330 PMC4498398 25614464

[8] Wang JC, Hsu PC, Wang KA, Wu WT, Chang KV (2023) Comparative effectiveness of corticosteroid dosages for ultrasound-guided glenohumeral joint hydrodilatation in adhesive capsulitis: a randomized controlled trial. Arch Phys Med Rehabil 104(5):745–752. 10.1016/j.apmr.2022.11.007 36521580 10.1016/j.apmr.2022.11.007

[9] Wu WT, Lin CY, Shu YC, Chen LR, Özçakar L, Chang KV (2023) Subacromial motion metrics in painful shoulder impingement: a dynamic quantitative ultrasonography analysis. Arch Phys Med Rehabil 104(2):260–269. 10.1016/j.apmr.2022.08.010 36055380 10.1016/j.apmr.2022.08.010

